# Gradient-Free Filter Importance for Structured Pruning of Compact EEG Networks †

**DOI:** 10.3390/s26196085

**Published:** 2026-09-25

**Authors:** Chu-Hui Lee, Chun-Ming Huang

**Affiliations:** 1 Department of Information Management, Chaoyang University of Technology, Taichung 413310, Taiwan; 2 Taiwan Semiconductor Research Institute, National Institutes of Applied Research, Hsinchu 300091, Taiwan; cmhuang@niar.org.tw

**Keywords:** EEG, brain–computer interface, EEGNet, structured pruning, filter importance, gradient-free pruning, model compression, quantization

## Abstract

Deploying EEG classification networks on resource-constrained brain–computer interface (BCI) hardware motivates structured filter pruning, yet which importance criterion to trust in already-compact architectures is unclear. We present a leakage-free, physically materialized comparison of five filter-importance criteria—ablation-based structural sensitivity analysis (SSA), first-order Taylor, L1-norm, geometric median, and random—across four datasets, two paradigms (motor imagery and ERP), two architectures, multiple capacities, and with and without fine-tuning. On EEGNet, after fine-tuning, SSA and Taylor are statistically equivalent: pooled over the four datasets their accuracy difference falls within a ±1 percentage-point margin (TOST, p=0.01), and on ShallowConvNet SSA is never worse than Taylor; both criteria significantly outperform the weight-based and random ones. SSA’s value is therefore not a more accurate ranking but its formulation: on a weight-quantized (8-bit) EEGNet, where the loss gradient—and hence Taylor—is undefined on the quantized graph used here, SSA is the best gradient-free criterion, significantly beating L1, geometric-median, and random pruning in aggregate—extending structured pruning to the quantized, gradient-free but editable deployment regime. We further show that the pruning schedule and fine-tuning matter more than the choice between strong criteria, and that training large then pruning with fine-tuning recovers accuracy equivalent to a directly trained smaller model. The results clarify where the importance criterion does and does not matter for compact EEG decoders.

## 1. Introduction

Electroencephalography (EEG)-based brain–computer interfaces (BCIs) decode neural activity for motor rehabilitation, assistive communication, and human–machine interaction [1,2]. Where classical pipelines relied on hand-crafted spatial filters such as CSP and FBCSP [3,4], deep learning now dominates EEG decoding [5,6], and compact convolutional networks such as EEGNet [7] and ShallowConvNet [8] have become standard decoders because they reach competitive accuracy with only a few thousand parameters. This compactness also makes them attractive targets for deployment on embedded and wearable BCI hardware, where memory, latency, and energy are constrained. Even at these small sizes, however, trained EEG CNNs contain filters that contribute little to the decision, and removing them through structured filter pruning—which deletes whole convolutional channels and leaves a dense, hardware-friendly network—can reduce cost further.

The difficulty is knowing *which* filters to remove. Most pruning criteria rely on indirect proxies for a filter’s importance: its weight magnitude [9,10], a first-order gradient (Taylor) estimate of its effect on the loss [11,12], or a geometric-redundancy measure [13]. A direct alternative is to measure importance by ablation: structural sensitivity analysis (SSA) [14] disables each filter, re-evaluates the model, and uses the measured accuracy drop as its importance. Whether such a direct, gradient-free measurement actually yields better pruning than the indirect proxies—in already-compact backbones, under a rigorous, leakage-free protocol, and across architectures and paradigms—has not been established.

This paper addresses that question. Our goals are (i) to compare SSA against the strong gradient- and weight-based importance criteria under a rigorous protocol; and (ii) to determine where, if anywhere, the choice of criterion actually matters for compact EEG CNNs. Concretely, we make the following contributions:

- We evaluate importance criteria under a leakage-free sensitivity estimate (scored on a held-out split of the training session), physically *materialized* pruning (filters are removed and the network rebuilt, so parameter and MAC counts are real rather than masked), and a deterministic, statistically tested pipeline.
- We benchmark five criteria (SSA, Taylor, L1, FPGM, random) across four datasets, two paradigms (motor imagery and ERP), two architectures, multiple capacities, and with/without fine-tuning.
- We report an honest and, to our knowledge, novel finding for EEG pruning: SSA and Taylor are statistically indistinguishable in pruned accuracy after fine-tuning, so direct ablation does not outperform a first-order gradient score here; both, however, clearly beat weight-based and random pruning.
- We reposition SSA’s value from accuracy to *applicability*: On a weight-quantized (8-bit) model, where the loss gradient (and thus Taylor) is undefined on the quantized graph, SSA is the best-performing gradient-free criterion—in the pooled four-dataset analysis it is significantly ahead of the cheap weight-geometry criteria L1 and FPGM—extending structured pruning to a gradient-free but editable regime relevant to embedded BCI deployment.
- We show that the pruning *scheduling* (per-layer vs. global) matters as much as the criterion, and that a set-large-then-prune-and-fine-tune workflow recovers equivalent accuracy to a directly trained small model, giving practical guidance when the right model size is not known in advance.

## 2. Related Work

**Compact EEG decoders.** End-to-end CNNs have largely replaced hand-crafted feature pipelines [3,4] for EEG decoding [5,6]. EEGNet [7] uses depthwise and separable convolutions to reach a few-thousand-parameter model competitive across BCI paradigms; DeepConvNet and ShallowConvNet [8] are widely used alternatives, and inception-style variants such as EEG-Inception [15] have been proposed for ERP decoding. The design space remains active—recent work explores multi-branch CNN–LSTM hybrids for cross-subject motor imagery [16] and transformer-based architectures [17]—but the compact convolutional backbones above are still the standard for resource-constrained BCI. Their compactness is precisely what makes further compression both desirable (for embedded deployment) and non-trivial (little obvious redundancy).

**Model compression for EEG.** Within EEG decoding, compression has been pursued mainly through quantization [18,19] and architectural redesign—for example, Q-EEGNet [20], an 8-bit EEGNet on an ARM Cortex-M microcontroller reaching large memory savings at a small accuracy cost—and, more broadly, knowledge distillation [21]. A related line reduces the *input* dimension rather than the network—for instance, efficient-attention EEG decoders that jointly perform channel selection [22], which is pruning-adjacent but operates on electrodes, not filters. The use of filter-level pruning in EEGs has received comparatively little attention, and existing studies typically apply generic magnitude- or gradient-based criteria without asking whether these indirect indicators remain reliable in already-compact backbones. Our work is complementary to quantization: we show that pruning and quantization compose, and in fact use a quantized model as the setting where our criterion’s gradient-free property becomes decisive.

**Structured filter pruning.** Pruning dates back to Optimal Brain Damage [23] and remains an active area with a wide range of methods [24], including recent compression frameworks that combine pruning with knowledge distillation under ordinal-consistency constraints [25]. Structured pruning removes entire filters, keeping dense convolutions that run efficiently on commodity hardware; unlike unstructured weight pruning [9], which needs sparse kernels. Importance criteria fall into a weight-based family—magnitude pruning [9] and L1-norm filter pruning [10]—a redundancy- or reconstruction-based family—for example, geometric-median pruning (FPGM) [13], channel pruning [26], ThiNet [27], and HRank [28]—and a gradient-based family that approximates each filter’s contribution to the loss via Taylor expansions [11,12]. Feature-map-rank criteria (HRank) are effective on image CNNs but degenerate on EEGNet—its feature maps are either full-rank or rank-collapsed to one, so every filter in a layer receives an identical rank; so, we exclude it. The gradient family is the closest prior method conceptually: like SSA it seeks each filter’s effect on the objective, but it *approximates* that effect through the loss gradient, whereas SSA *measures* it by re-evaluation. Whether pruning a large model outperforms training the target-size model directly [29,30] we examine in the EEG-CNN setting. Relative to all of these, our contribution is not a new criterion but a rigorous, honest characterization of when ablation-based and gradient-based importance differ in accuracy (they do not differ, after fine-tuning) and in applicability (they differ on gradient-free models).

## 3. Materials and Methods

### 3.1. Problem Formulation and Overview

We study *structured filter pruning* of compact EEG convolutional networks: given a trained network with a set of prunable convolutional filters, remove a fraction of them so that a physically smaller network is obtained while classification accuracy is preserved. The central question is: How does ablation-based importance (SSA) [14] compare against the standard weight- and gradient-based importance criteria across architectures, datasets, and experimental paradigms under a leakage-free and physically materialized protocol?

The pipeline is as follows: (i) train a baseline network; (ii) score every prunable filter by an importance criterion; (iii) select the lowest-importance filters under a scheduling policy; (iv) materialize the reduced network (physically remove filters and rebuild the graph); and (v) evaluate, optionally after a short fine-tune. All design choices below are shared by every criterion so that the *only* difference between methods is the per-filter score.

### 3.2. Importance Criteria

For a trained model with parameters θ and prunable filters $C=\{c_{1},\ldots ,c_{n}\}$, each criterion assigns a scalar importance to every filter; the lowest-importance filters are removed first.

- **SSA (ours).** Ablation-based sensitivity: The output channel of filter $c_{i}$ is *temporarily masked* (its output set to zero via a forward hook) and the model is re-evaluated, giving $S_{i}=A\left(\theta \right)-A\left(\theta \setminus c_{i}\right)$, the resulting drop in accuracy. A large $S_{i}$ marks a task-critical filter; near-zero or negative $S_{i}$ marks a removable one. Because this masking precedes batch normalization it is a *proxy* for the effect of physically removing the filter rather than an exact measure of it (Section 3.4); we use the masked score only to *rank* filters, and every reported pruned model is physically materialized. SSA is directly accuracy-based and gradient-free, so it can score filters even where no usable loss gradient exists—most notably on post-training-quantized networks—unlike Taylor and other gradient-based criteria (Section 5.4).
- **Taylor [11].** First-order estimate of the removal effect on the loss, $\left|\sum_{w\in c_{i}}w\cdot \partial L/\partial w\right|$, obtained from one forward–backward pass.
- **L1 [10].** The L1 norm of the filter weights (data-free).
- **FPGM [13].** Geometric-median redundancy: filters close to the others (small summed pairwise distance) are the most redundant (data-free).
- **Random.** Uniform random selection at matched counts (10 seeds); used as a control.

The criteria differ in cost and assumptions: Taylor, L1, and FPGM are cheap (one pass or data-free) but indirect (Taylor is a first-order approximation of the loss change; L1/FPGM are weight-geometry proxies), whereas SSA is direct and gradient-free but requires one forward evaluation per filter and inherits the resolution of the validation set used for scoring. These four are established, widely adopted representatives of the weight-, redundancy-, and gradient-based families of importance criteria, chosen as strong and reproducible baselines rather than as the most recent state of the art; a comparison against newer structured-pruning methods (e.g., high-rank feature-map pruning or dependency-graph approaches) is left to future work. Taylor, being the strongest of these baselines in our experiments, serves as the primary point of comparison for SSA.

*Computation details (for reproducibility).* All scores are computed on the trained model *once* (one-shot; not recomputed after each removal) and on the same held-out split (Section 3.3), with the model in evaluation mode—batch-normalization running statistics frozen and dropout disabled—so that scoring is deterministic. For SSA, each prunable output channel is zeroed in turn by a forward hook (before the subsequent batch-normalization and nonlinearity) and the network is re-evaluated over the entire split; the sensitivity is the resulting drop in accuracy, $S_{i}=A\left(\theta \right)-A\left(\theta \setminus c_{i}\right)$. Because the validation split is small (∼57 trials on IV-2a), the finest resolvable accuracy change is 1/57≈1.8 points, so many filters share identical scores; ties are broken *deterministically* by a stable sort on $\left(S_{i},\mathrm{layer}\;\mathrm{index},\mathrm{channel}\;\mathrm{index}\right)$, making the selected set reproducible. For Taylor, a single pass over the split accumulates, per filter, $\left|\sum_{w\in c_{i}}w\;\partial L/\partial w\right|$—the absolute value is taken *after* summing the signed weight–gradient products over the filter (weight terms only), and the per-filter scores are averaged over mini-batches. L1 and FPGM are computed directly from the weights and use no data. For every criterion the lowest-scoring filters are removed first under the scheduling policy of Section 3.6.

### 3.3. Leakage-Free Sensitivity Estimation

Sensitivity, and every importance score, must not be computed on the data used for final evaluation. Scoring filter importance on the evaluation session (session-E) would be a leakage confound: the retained filters are selected precisely to preserve accuracy on the trials that subsequently score the result, which can inflate the apparent quality of any importance criterion. We therefore adopt a strictly leakage-free protocol: every importance score is computed on a held-out validation split of the *training* session (session-T, 80/20 split, seed 42)—the trials the model never saw—while the evaluation session (session-E) is reserved strictly for the final reported accuracy, so selection and evaluation never share data. Concretely, for each subject the four data uses are disjoint by construction: the baseline is *trained* on the session-T training portion; every filter is *scored* on the session-T held-out validation split; the pruned network is *fine-tuned* on the same session-T training portion (fixed 50-epoch budget, no validation-based checkpoint selection on the pruned model, so the scoring split is never used for model selection); and all reported *accuracy* is measured on session-E. No quantity computed on session-E ever enters scoring, pruning, or fine-tuning.

### 3.4. Materialized Pruning

A pruned filter must be physically removed, not merely masked. Masking (multiplying a Conv2d output channel by zero via a forward hook) is invalid for efficiency claims: batch normalization converts a zeroed channel into a non-zero constant, so a masked filter still contributes downstream and the parameter/MAC counts are fictitious. We instead materialize every pruned network: surviving filters are physically copied into a smaller network and removed filters are deleted, so tensor shapes actually shrink. For EEGNet, the grouped depthwise convolution couples the temporal and spatial filters; we handle the resulting unequal groups with an input-channel *gather* (no extra parameters), so any per-filter removal set yields a valid reduced network.

Concretely, let keep_t be the surviving temporal channels ($\left|\mathrm{keep}\text{\_}t\right|=k_{t}\leq F_{1}$) and keep_mid the surviving middle channels ($\left|\mathrm{keep}\text{\_}\mathrm{mid}\right|=k_{\mathrm{mid}}\leq F_{1}D$). Because each middle channel *j* is driven by temporal parent ⌊j/D⌋, an uneven removal leaves parents with different numbers of surviving children, which a single grouped Conv2d cannot express. The materializer sidesteps this by emitting a *fully* depthwise convolution (groups $=k_{\mathrm{mid}}$) preceded by a parameter-free channel-routing step (Algorithm 1).
**Algorithm 1:** Coupled group-convolution materialization for EEGNet.**Input:** trained temporal weights $W_{t}\in R^{F_{1}\times 1\times 1\times T}$ and depthwise weights $W_{d}\in R^{F_{1}D\times 1\times C\times 1}$; surviving temporal indices $K_{t}$ and middle indices $K_{m}$ ($k_{t}=\left|K_{t}\right|\leq F_{1}$, $k_{\mathrm{mid}}=\left|K_{m}\right|\leq F_{1}D$).*Build reduced weights:*   1.     $W_{t}^{\prime }\leftarrow W_{t}\left[K_{t}\right]$ (surviving temporal filters; shape $k_{t}\times 1\times 1\times T$).   2.     $W_{d}^{\prime }\leftarrow W_{d}\left[K_{m}\right]$ (surviving middle filters; shape $k_{\mathrm{mid}}\times 1\times C\times 1$).   3.     routing map $g\left[j\right]\leftarrow \mathrm{pos}\;\left(K_{t},\left\lfloor K_{m}\left[j\right]/D\right\rfloor \right)$ for $j=1,\ldots ,k_{\mathrm{mid}}$ (index of each survivor’s temporal parent; carries no parameters).*Forward pass* (input $X\in R^{N\times 1\times C\times T}$):   4.     $H\leftarrow \mathrm{BN}\;\left(\mathrm{Conv}2d(X,W_{t}^{\prime })\right)$ ($N\times k_{t}\times C\times T$).   5.     $H^{\prime }\leftarrow H\left[:,g,:,:\right]$ (channel gather routes each surviving middle channel to its surviving temporal parent; $N\times k_{\mathrm{mid}}\times C\times T$).   6.     $Y\leftarrow \mathrm{Conv}2d\;\left(H^{\prime },W_{d}^{\prime },\;\mathrm{groups}=k_{\mathrm{mid}}\right)$ ($N\times k_{\mathrm{mid}}\times 1\times T$).**Output:** reduced network producing Y. Because the gather carries no learnable weights, parameters and MACs strictly decrease for any non-empty removal set.

The gather step carries no learnable weights, so the reduced network has strictly fewer parameters and MACs than the original for any non-empty removal set, and the grouped-conv group-equality constraint is satisfied by construction. Materialization is verified by a gate suite (bit-exact identity when nothing is removed; output invariance to the removed filters’ weights; and a demonstration that masked ≠ materialized because of the batch-norm leakage). Efficiency (parameters, multiply–accumulates via fvcore with a thop cross-check < 1%) is computed on these physically reduced networks. To be explicit about the two roles of zeroing a channel: zeroing is used *only* as a transient probe when *scoring* a filter’s sensitivity (Section 3.2); every *reported* pruned model—and all accuracy, parameter, and MAC numbers—uses physical materialization, never a masked network. The two are not interchangeable: on IV-2a a masked subnetwork and the corresponding materialized subnetwork differ in test accuracy by up to several points at aggressive ratios (and the masked network’s “parameters” are unchanged from baseline), which is why all the results in this paper are materialized.

### 3.5. Post-Training Weight Quantization

For the gradient-free deployment scenario (Section 5.4) we simulate the weight path of standard post-training quantization (PTQ). Each convolution and linear weight tensor w (every parameter of rank ≥2) is quantized per-tensor and symmetrically to 8 bits,

(1) $$q\left(w\right)=\mathrm{round}\left(w/s\right)\cdot s,\;s=\max|w|/\left(2^{7}-1\right),$$

with w/s clamped to [−127,127], before rescaling. The scale *s* is taken from the tensor’s own maximum magnitude, so no calibration data—and in particular no test data—enter the quantizer. Biases and all activations are kept in floating point and batch-normalization parameters are not folded, so this is a weight-quantized *simulation* rather than a fully integer-executed graph; that is sufficient for our purpose, which is a graph on which the loss-to-weight gradient is unavailable (a genuine int8 runtime has no autograd at all). Quantization is applied to the trained model *before* pruning; SSA, L1, and FPGM then score filters on the quantized weights, and the materializer (Algorithm 1) copies the surviving quantized weights into the smaller network—we do not re-quantize or fine-tune afterwards. Because round(·) has zero derivative almost everywhere, the gradient of the loss with respect to w through *q* is identically zero (measured norm 0.00 versus 5.0 on the float graph), which is precisely why a Taylor score cannot be formed on this graph, whereas SSA, needing only forward evaluations, can.

### 3.6. Scheduling and the Removal Floor

Two schedules are compared for every criterion. Per-layer removes the lowest-importance fraction *p* within each prunable layer independently. Global pools all filters into one ranking and removes the lowest fraction *p* overall, letting redundant layers absorb a larger share of the cut. A per-layer *floor* keeps at least one filter per layer, and—for architectures with cross-layer coupling—a validity guard skips any removal that would empty a coupled layer, so aggressive ratios remain materializable. Under the global schedule the scores are pooled *without* cross-layer re-normalization. This is well-defined for SSA because its score is a measured accuracy drop, expressed in the *same units across all layers*; for the weight- and gradient-based scores (L1, FPGM, Taylor) the raw magnitudes are not directly comparable across layers, so a global ranking would be biased toward the layer with the largest raw scores. For this reason the per-layer schedule is the common default applied to every criterion in the main comparison, and the per-layer-vs-global contrast is examined—and reported in the six-method benchmark (Table 3)—only for SSA, whose same-unit score makes global pooling meaningful.

### 3.7. Statistics

The method comparisons use paired two-sided *t*-tests across subjects, with Wilcoxon signed-rank tests as a non-parametric check and Cohen’s $d_{z}$ for paired effect size; 95% confidence intervals are reported for accuracy differences and, where a family of comparisons is made, Holm correction is applied. For the class-balanced P300 set, where balanced accuracy coincides with accuracy, we additionally report threshold-independent ROC-AUC. The deterministic recipe (fixed seeds, deterministic cuDNN, CUBLAS workspace configuration) makes every run bit-reproducible within a fixed hardware and software environment (the exact GPU, CUDA/cuDNN and library versions are reported with the released code); exact bit-level values may differ on other stacks.

### 3.8. Training Regimes

Two regimes are used and stated explicitly. The benchmark experiments (Section 4.1) use a longer recipe (Adam, lr $1.5\times 10^{-3}$, up to 1000 epochs, patience-based early stopping on the training loss, last-epoch checkpoint), giving the reference EEGNet-8,2 baseline of 66.3% on IV-2a. The capacity study (Section 4.5) instead uses one unified recipe (500 epochs, lr $1\times 10^{-3}$, batch 64, best-validation checkpoint) across all capacities for a fair cross-capacity comparison, giving a slightly lower EEGNet-8,2 baseline (≈60% on IV-2a); a control varying recipe and checkpoint attributes the gap to the optimization recipe (epoch budget, learning rate, batch size), not to reduced training data (both regimes hold out the same leakage-free split). This absolute level is immaterial to our conclusions: every pruning comparison is made *within a fixed baseline model* (paired differences relative to that model’s own accuracy), so the baseline level cancels. Fine-tuning, when applied, uses 50 epochs at lr $1\times 10^{-4}$.

### 3.9. Architectures, Datasets, and Protocol

We use two compact EEG CNNs: EEGNet (8,2 configuration; 3932 parameters) as the primary backbone, and ShallowConvNet as a second, structurally different architecture. Four datasets across two paradigms are used (Table 1): three motor imagery sets— BCI IV-2a [31,32] (4 classes, 22 channels, 9 subjects), BCI IV-2b [33] (2 classes, 3 channels, 9 subjects), HGD [8] (4 classes, 44 channels, 14 subjects)—and one event-related potential set, BNCI2014-009 [34] (P300, balanced 2 classes, 16 channels, 10 subjects), accessed through the MOABB benchmark [35] and MNE [36]. All signals are processed to common 250 Hz, 1 s–4 s trial windows as appropriate to the paradigm; the P300 set is class-balanced by subsampling non-targets so that accuracy remains a meaningful metric. Evaluation is within-subject (train session → test session). All models are implemented in PyTorch (v2.11.0) [37]; multiply–accumulate counts use fvcore with a THOP cross-check.

The per-dataset preprocessing is summarized in Table 2. Every dataset is epoched with the MOABB default pipeline for its paradigm, band-pass filtered, resampled to a common 250 Hz, and used without additional artifact rejection or per-channel normalization; the same fixed 80/20 split of the training session (seed 42) provides the leakage-free validation set on which all importance scores are computed, while the separate evaluation session is used only for the reported test accuracy.

## 4. Results

### 4.1. Primary Benchmark (EEGNet × IV-2a)

The leakage-free sensitivity scores are strongly right-skewed (Figure 1a): a large fraction of filters have near-zero or slightly negative sensitivity (the slightly negative values lie within the resolution of the small validation set and are treated as removable rather than read as genuine accuracy gains), confirming substantial removable redundancy even in a 3932-parameter backbone. Sensitivity is concentrated in the early (temporal, depthwise) layers, while the separable-pointwise layer is almost uniformly low-sensitivity (Figure 1b)—the main pool of removal candidates.

Table 3 and Figure 2 report the six criterion–schedule configurations (the five criteria, with SSA evaluated under both per-layer and global scheduling) at pruning ratios 0.1–0.4 (materialized, no fine-tune, nine-subject mean; baseline: 0.663). SSA-per-layer and Taylor are the two strongest criteria at every ratio (e.g., 0.541/0.541 at r=0.2, 0.422/0.434 at r=0.4), followed by SSA-global; L1, FPGM, and random trail, with FPGM weakest (0.307 at r=0.4). Both leading criteria clearly separate from random pruning, confirming that the importance ranking carries real information. On real (materialized) hardware metrics, SSA-per-layer reduces parameters from 3932 to 2863 (−27%) and MACs from 22.9 M to 17.0 M (−26%) at r=0.2 (Figure 3).

Scheduling matters as much as the criterion (Figure 4). Global pruning concentrates the cut in the low-sensitivity pointwise layer and spares conv_temporal; once that reserve is exhausted, it must cut task-critical filters, so per-layer ≫ global at aggressive ratios (SSA: 0.422 vs. 0.368 at r=0.4). We therefore report per-layer as the headline schedule.

### 4.2. SSA vs. Taylor: A Parity, Not a Superiority

Because SSA and Taylor are consistently the two strongest criteria, we test them head-to-head with paired statistics across subjects, per dataset and ratio, both without and with fine-tuning (Table 4, Figure 5). Under fine-tuning the two are statistically indistinguishable at the aggregate level: every ratio-averaged difference is non-significant (IV-2a: +0.005, IV-2b: +0.003, HGD: −0.004, ERP: −0.007), and among the 24 fine-tuned dataset × ratio cells only one (ERP at ratio 0.3, −0.010) reaches nominal significance (p=0.019), which does not survive Holm correction for multiplicity. Because a non-significant difference is not by itself evidence of equivalence, we additionally apply two one-sided tests (TOST). We fix the equivalence margin at ±1 percentage point a priori: a difference below one accuracy point is within run-to-run variation for these datasets and is not operationally meaningful for a BCI decoder. Pooling all four datasets (42 subjects), the fine-tuned SSA –Taylor difference (mean: −0.001) is statistically equivalent within the ±1 percentage-point margin ($p_{\mathrm{TOST}}=0.010$; and within ±2 points at $p<10^{-3}$). We note that this equivalence is a pooled result: per dataset the differences are small and non-significant but the samples (n=9–14) are underpowered for a formal equivalence claim (per-dataset TOST p=0.10–0.29), so we present ±1-point equivalence as a pooled finding, consistent but individually underpowered on each dataset.

To treat both settings symmetrically, we apply the identical TOST without fine-tuning. There, the two are *not* equivalent within ±1 point (pooled $p_{\mathrm{TOST}}=0.97$): Taylor holds a small but real edge (pooled SSA–Taylor =−0.032; e.g., IV-2a: −0.033, p=0.06; HGD: −0.060, p=0.06; ERP: −0.021, p=0.03). The symmetric analysis thus makes the mechanism explicit: fine-tuning is what brings the two criteria into formal equivalence; before it, the smooth gradient score is modestly better. Either way, SSA does not outperform the strongest gradient criterion after fine-tuning; its value is that it *matches* it—to within one accuracy point—while being gradient-free, directly accuracy-based, and interpretable.

While SSA and Taylor are equivalent to *each other*, both remain clearly superior to the weight-based, redundancy-based, and random criteria, and this separation is statistically robust across datasets. Pooled over the four datasets (42 subjects; Table 5), SSA and Taylor each significantly outperform L1, FPGM, and random, both without and with fine-tuning (all: p<0.001). Fine-tuning shrinks every gap—for example, SSA−L1 falls from +0.077 (no-FT) to +0.026 (+FT)—but none closes, confirming that the criterion still matters relative to the weak baselines even after retraining, whereas the SSA/Taylor distinction does not.

### 4.3. Cross-Paradigm Robustness (MI vs. ERP)

The SSA–Taylor relationship is paradigm-independent: the ERP dataset shows the same pattern as the three motor-imagery sets—indistinguishable under fine-tuning (overall, −0.007, ns; Table 4), and Taylor marginally ahead without it. The parity finding therefore does not depend on the decoding paradigm.

Because the BNCI2014-009 P300 test set is class-balanced by construction (96 target/96 non-target trials per subject), balanced accuracy is numerically identical to accuracy here (they agree to machine precision), so it adds no information; we therefore additionally report the threshold-independent ROC-AUC, the standard operating-point-free metric for P300. AUC preserves the accuracy-based ordering (+FT, ratio-averaged over the 10 subjects): SSA (0.925) and Taylor (0.930) are the top pair and remain mutually indistinguishable (ΔAUC=−0.004, p=0.09), both above FPGM (0.917), L1 (0.912), and random (0.905). SSA and Taylor each beat random significantly ($p\leq 10^{-3}$); as with accuracy, their individual-dataset margins over L1 and FPGM are positive but reach significance only in the pooled four-dataset analysis (Table 5). The criterion pattern is thus not an artifact of the 0.5 decision threshold.

### 4.4. Multi-Architecture Generalization (ShallowConvNet)

Repeating the six-method benchmark on ShallowConvNet across all four datasets (Figure 6, Table 6), evaluated without fine-tuning, reproduces the EEGNet pattern: SSA and Taylor are top-tier and statistically indistinguishable (all four datasets: ns; overall differences: −0.010 to −0.019), both beat the weight-based and random criteria, and per-layer again dominates global at aggressive ratios (e.g., HGD: 0.613 vs. 0.386 at r=0.4). The criterion ranking is thus stable across a structurally different backbone.

To test the two top criteria on this second architecture *with* fine-tuning as well, we repeated the SSA-vs.-Taylor comparison on ShallowConvNet × IV-2a with the same 50-epoch fine-tuning and leakage-free scoring (nine subjects, per-layer, ratios 0.1–0.6). After fine-tuning, SSA is at least as accurate as Taylor on this architecture: the paired difference is positive at every ratio, small and non-significant in the mild-pruning regime relevant in practice (+0.008 to +0.012 at ratios 0.1–0.4; all ns), and larger at aggressive ratios (+0.031 at r=0.5, +0.073 at r=0.6), giving a ratio-averaged +0.024 (90% CI [+0.011,+0.037], p=0.010). Consequently, a ±1-point TOST does not certify equivalence on ShallowConvNet—but here the departure is in SSA’s favour, not against it. We therefore treat the formal ±1-point SSA–Taylor *equivalence* as an EEGNet-specific result (Section 4.2); on ShallowConvNet the robust statement is that SSA is never worse than Taylor after fine-tuning, with a modest edge that emerges only at high pruning ratios on this single dataset and should not be over-generalized.

### 4.5. Train-Large-Then-Prune (Capacity Study)

Pruning is often motivated by not knowing the right model size a priori: one sets the network large and prunes down. We test this directly. Directly trained EEGNet accuracy increases with capacity on the harder sets (IV-2a: 0.580 → 0.637 from $F_{1}=4$ to 32; HGD: 0.822 → 0.901) and saturates on the easy 2-class sets (IV-2b, ERP), motivating “set large” where capacity helps (Figure 7). We then prune the largest model (EEGNet-32,2) down across ratios and compare, at matched parameter counts, against directly trained smaller models (Figure 8). With fine-tuning, pruning the large model recovers the directly trained small model’s accuracy across all datasets (within ≈1–3 points, occasionally exceeding it), whereas the training-free (no-fine-tune) subnetwork is substantially worse. Among criteria, the parity persists: SSA and Taylor track each other, both above the weight/random baselines, and per-layer above global (Figure 9). “Set-large-then-prune-and-fine-tune” is therefore a viable substitute for choosing the size in advance.

## 5. Discussion

### 5.1. From “A Better Criterion” to “When the Criterion Matters”

A direct ablation measurement might be expected to prune more accurately than indirect gradient or weight approximations. Comparing SSA against those criteria under a rigorous protocol puts that expectation to the test, and the result is nuanced. Across four datasets, two paradigms, two architectures, and a range of pruning ratios, ablation-based sensitivity (SSA) and first-order gradient importance (Taylor) are the two strongest criteria and, after fine-tuning, are statistically equivalent rather than merely non-different: pooled over the four datasets the SSA–Taylor accuracy difference lies within a ±1 percentage-point equivalence margin (two one-sided tests, p=0.01; Section 4.2), and the same top-tier, mutually indistinguishable pattern reappears on a second, structurally different backbone (ShallowConvNet, evaluated without fine-tuning). The paired SSA–Taylor differences straddle zero at every pruning ratio (Figure 5); without fine-tuning, Taylor is, if anything, marginally ahead on the harder datasets. Direct ablation therefore does not, in these compact EEG networks, yield more accurate pruning than a first-order Taylor score—a parity, not a superiority.

Why do a measured ablation and a first-order approximation reach the same accuracy? Not because they agree on which filters to remove—they do not. Ranked filter-by-filter, SSA and Taylor correlate only modestly (per-layer Spearman 0.24–0.48 across the four datasets) and the sets they actually cut overlap by only 28–36% (Jaccard), yet their fine-tuned accuracies are statistically identical. Three measured facts explain this (Figure 10). First, the accuracy is largely *insensitive* to *which* low-importance filters are removed: two prunings that share barely a third of their cuts converge to the same accuracy once the surviving network is briefly retrained, so there is little headroom for a “smarter” ranking to capture—exactly why the ±FT contrast (Section 5.3) dwarfs the SSA–Taylor contrast. Second, among the cheap criteria Taylor is nonetheless the closest surrogate to ablation: SSA correlates about twice as strongly with the Taylor gradient as with weight-magnitude L1 (e.g., 0.24 vs. 0.14 on IV-2a), which is why SSA and Taylor form the joint top pair while L1 and FPGM trail. Third, SSA’s one distinctive lever—directly measuring task accuracy—is resolution-limited on realistic EEG validation sets: 26–50% of filters change validation accuracy by less than a single trial and are therefore unrankable by SSA (worst in the pointwise separable convolution, up to 77%), and the SSA–Taylor agreement rises monotonically with validation-set size (Spearman: 0.24 at 57 trials to 0.48 at 160), the signature of a small-sample resolution limit rather than a genuine ranking gap. Directness thus buys a fidelity that the compact, fine-tuned network has almost no accuracy to spend—consistent with the κ-sensitivity control of Section 5.4. Parity, in other words, is what a compact, fine-tuned EEG-CNN pipeline should produce.

This does not weaken the case for studying importance criteria in EEG pruning; it sharpens the question from *which criterion is best* to *when the choice of criterion matters at all*. Our answer has three parts: for accuracy, under fine-tuning, it essentially does not matter among the strong criteria (SSA ≈ Taylor, with both ≫ weight/random); the *schedule* matters more than the criterion (Section 5.2); and the one place the criterion is decisive is applicability, not accuracy (Section 5.4).

### 5.2. Scheduling Matters as Much as the Criterion

The allocation of the cut across layers is at least as important as how filters are ranked. Per-layer pruning, which enforces the same ratio in every layer, consistently tolerates more aggressive pruning than a global schedule that pools all filters into one ranking. The gap is small at mild ratios but large at aggressive ones and grows with task difficulty: at a 0.6 ratio (with fine-tuning) per-layer versus global accuracy is 0.576 vs. 0.486 on IV-2a, 0.826 vs. 0.666 on HGD, and 0.839 vs. 0.752 on the ERP set, while on the easy IV-2b set the two are indistinguishable (0.723 vs. 0.724). The mechanism is visible in the allocation maps: global pruning spends the abundant low-sensitivity filters of the pointwise layer first and, once that reserve is exhausted, is forced to cut into task-critical early layers; per-layer pruning never over-drains a single block. Because this holds for every criterion and across datasets, paradigms, and architectures, we conclude that scheduling is a first-level design choice in EEG pruning, not a detail.

### 5.3. Train-Large-Then-Prune, and the Necessity of Fine-Tuning

Pruning is often motivated by not knowing the right model size in advance: one sets the network large and prunes down. Our capacity study supports this workflow but with an important qualification. Directly trained accuracy increases with capacity where the task has headroom (IV-2a: 0.600 → 0.637 from EEGNet-8,2 to -32,2; HGD: 0.864 → 0.901) and saturates where it does not (the easy 2-class IV-2b and ERP sets). Pruning the largest model back down and fine-tuning recovers the accuracy of a directly trained smaller model of matched *parameter budget* (the directly trained references vary width through $F_{1}$, so this is a parameter-budget rather than an architecture-controlled comparison), across all datasets, and at the source capacity even slightly exceeds it. The training-free (no-fine-tune) subnetwork, however, is markedly worse. Set-large-then-prune is therefore a viable substitute for choosing the size a priori, but only in combination with fine-tuning—a result consistent with the broader “rethinking pruning” literature and here confirmed in the EEG-CNN setting. Within this study, the criterion parity persists: SSA and Taylor track each other along the accuracy–parameter frontier, both above the weight and random baselines. Viewed in this way, structured pruning is a restricted, removal-only form of architecture search over channel widths [29]: the useful output is the resulting per-layer width configuration rather than the inherited weights. We do not pursue a full architecture search here—that would require comparison against dedicated search methods and is beyond the scope of a criterion benchmark—but the framing clarifies why the schedule (which allocates widths across layers) matters as much as the ranking criterion.

This width-configuration view also bounds how far SSA’s O(n)-forward cost can be amortized. If a single subject-agnostic ranking pruned every subject well, the scoring cost would be paid once; it does not. Filter importance is strongly subject-specific: the per-filter SSA scores of different subjects are essentially uncorrelated (mean pairwise Spearman between −0.03 and +0.00 across the four datasets), and a leave-one-subject-out *consensus* ranking—the mean SSA score of all other subjects, applied to the held-out subject—prunes significantly worse than that subject’s own ranking on every dataset (accuracy deficit of +0.05 to +0.11, all p≤0.001, no fine-tuning; Figure 11). The consensus ranking is no better than random on IV-2a and IV-2b, and only partially recovers on the larger HGD and ERP sets, so a reused *mask* does not transfer. What is stable across subjects is the coarser signal the architecture-search view privileges: the per-layer *allocation* of the cut (every subject protects the temporal convolution and concentrates removal in the separable layers). Amortization in EEG pruning is therefore better sought at the level of a transferable width recipe—reused, then trained per subject—than by reusing a filter ranking, whose value is inherently per-subject.

### 5.4. Why an Ablation Criterion, Given Parity

Parity, and Taylor being the far cheaper score (one backward pass for all filters versus one forward per filter—about two orders of magnitude more ranking time on the source model, Table 7), raises the question of when SSA should be preferred. Two control experiments bound the answer, and we are deliberately narrow in our conclusions as a result.

First, SSA’s directness does *not* translate into better selections, even for a non-differentiable objective. BCI systems frequently report Cohen’s κ rather than accuracy; κ has no gradient, so Taylor can only differentiate the cross-entropy loss, whereas SSA can score filters by measured Δκ. One might expect an advantage here, but the data show the opposite: ranking by κ sensitivity does not improve pruned κ over Taylor (nine-subject mean pruned κ at r=0.2: 0.386 for SSA-κ vs. 0.451 for Taylor; overall: SSA-κ–Taylor =−0.063, p=0.06), and is even worse than ranking by accuracy sensitivity. The cause is resolution: κ estimated on a ∼57-trial validation set is high-variance, so directly measuring it yields a noisier ranking than Taylor’s smooth loss-gradient surrogate. We therefore do not claim that direct measurement produces better rankings; when a usable gradient exists, Taylor is an equally good and cheaper default. This also delimits the “directness” narrative for SSA.

The coarse resolution is, moreover, not fixable by a finer objective. Replacing SSA’s step-function accuracy target with a continuous one—the exact increase in cross-entropy loss, or the drop in mean true-class softmax probability, when a filter is ablated—removes the ties entirely (the fraction of unrankable, zero-sensitivity filters falls from ≈25% to 0 on IV-2a) and gives a strictly finer ranking. It does not improve pruned accuracy: ratio-averaged over the nine subjects (no fine-tuning), loss and probability sensitivity reach 0.491 and 0.513 versus 0.497 for accuracy sensitivity and 0.501 for Taylor—all statistically indistinguishable (p>0.2). Finer resolution thus buys ranking precision the redundant network cannot convert into accuracy: the bottleneck is the insensitivity of accuracy to *which* low-importance filters are removed (Section 5.1), not the coarseness of any single criterion. This brackets the κ control above—a rougher objective is worse, a finer one is no better—and it also shows the criteria’s rough agreement is not evidence that Taylor approximates the loss well: the exact loss sensitivity actually correlates *less* with Taylor (per-layer Spearman: 0.07) than accuracy sensitivity (0.24), because ablating an entire filter is a large perturbation a first-order score does not track.

Second, SSA’s genuine, and experimentally demonstrated, distinct value is *applicability where the loss gradient is undefined but the model is still editable*—together with the best pruned accuracy in that regime. The concrete case is structured pruning applied after post-training quantization, a standard step in deploying EEGNet-class models to microcontrollers. Weight-quantizing EEGNet to 8 bits leaves accuracy essentially unchanged (within 0.3 points of float on all four datasets), yet the Taylor score is undefined on the quantized graph: the quantizer rounding q(w)=round(w/s)·s has an identically zero gradient, so |w·∂L/∂w| collapses to exactly zero for every filter (measured gradient norm through the int8 quantizer: 0.00, versus 5.0 on the float graph) and no gradient-based ranking can be formed. A straight-through estimator only recovers a gradient by rebuilding a differentiable surrogate, i.e., by leaving the quantized model.

The weight-geometry criteria L1 and FPGM, however, remain computable on the quantized weights, so the decisive question is not whether SSA *can* rank filters there—L1, FPGM, and random can too—but whether its direct, task-performance-based score is *worth* its cost once the cheap gradient default (Taylor) is unavailable. It is. Pruning the quantized model with SSA gives the highest accuracy at every compression ratio on all four datasets (Figure 12), and pooled over the four datasets (42 subjects) SSA significantly outperforms L1 (+0.064, $p=4\times 10^{-4}$), FPGM (+0.057, $p<10^{-4}$), and random (+0.070, $p<10^{-6}$; Table 8). Because our materialization operates on the quantized weights directly, the chosen filters are physically removed to give a smaller quantized network.

When a float model *is* available, an alternative is to prune it with Taylor and *then* quantize. We compared the two pipelines—quantize then prune with SSA (Pipeline A), versus prune the float model with Taylor then quantize (Pipeline B)—across all four datasets (42 subjects, per-subject paired). The two are statistically indistinguishable when pooled (A−B =−0.014, p=0.28) and on three of four datasets individually (IV-2a: −0.026, p=0.38; IV-2b: +0.018, p=0.16; HGD: +0.001, p=0.98), while on ERP the float pipeline is better (−0.053, p=0.02). We therefore do *not* claim that operating on the quantized model is more accurate than the float pipeline; where a float model and its gradient are available, prune-then-quantize with Taylor is at least as good and sometimes better. SSA’s distinct value is specifically the case the float pipeline cannot serve: when *only* the quantized model is available and no gradient can be formed, SSA is the best-performing gradient-free option (significantly ahead of L1, FPGM, and random), with no statistically significant difference from the float pipeline on three of the four datasets.

We scope this claim carefully. Pruning requires an editable model: the removal step rebuilds the network and cannot be performed on an exported inference-only artifact (TFLite/ONNX), where neither SSA nor Taylor can prune. We therefore do not claim SSA prunes black-box models, only that it extends structured pruning to the quantized, gradient-free but editable setting gradient-based criteria cannot reach. Interpretability ($S_{i}$ is a measured drop in the target metric’s own units) is a secondary benefit.

SSA is the most expensive criterion—one forward evaluation per filter makes it about two orders of magnitude slower than a single-backward-pass Taylor score (∼140× here) and three orders of magnitude slower than the weight-only L1 norm—and on the float model (Table 4, Table 6) this buys no accuracy advantage after fine-tuning. Its distinguishing value appears only in the last column of Table 7: among the criteria that survive quantization (Taylor does not), SSA is the one whose direct, task-based score translates into the best pruned accuracy—in aggregate significantly ahead of the cheap weight-geometry criteria L1 and FPGM (Table 8)—so, it is preferable precisely, and only, in the gradient-free-but-editable deployment regime.

### 5.5. Robustness Observations

The specific set of removed filters is only weakly reproducible across source-model training budgets: for both SSA and Taylor, the overlap of removed filters between models trained for 100, 500, and 2000 epochs is near chance (Jaccard ≈ 0.17–0.20) while pruned accuracy varies comparably (std ≈ 0.05). This instability is not specific to SSA; it confirms that redundancy in these compact networks is diffuse; so, *how many* filters are removed matters more than *which*—consistent with the SSA–Taylor parity and its κ control above.

### 5.6. Limitations

Our conclusions are bounded in several ways. (i) we target *compact* EEG CNNs; SSA’s O(n)-forward cost and the diffuse redundancy we observe may not transfer to larger backbones. (ii) Importance is scored on small, subject-specific validation sets, whose coarse resolution limits direct-measurement criteria. (iii) The SSA ranking is subject-specific (Section 5.3, Figure 11), so cross-subject transfer, if any, must act at the coarser width-recipe level, which we do not evaluate. (iv) The gradient-free advantage uses weight-only 8-bit quantization and an editable model; full activation quantization and exported runtimes are out of scope. (v) DeepConvNet and an EEG-Inception variant were not competitive in our setup and were excluded; their inclusion and an SSVEP paradigm are natural extensions. (vi) A feature-map-rank criterion (HRank) degenerates on EEGNet (Section 2); comparison against other recent structured-pruning methods remains future work. (vii) Efficiency is measured as parameters and MACs, not on-device latency, memory, or energy; an embedded benchmark is left to future work. (viii) The train-large-then-prune comparison matches models by parameter budget; training the pruned width from scratch, to separate weights from widths, is a natural next step.

These recent families—dependency-graph tools that decide *how* to remove coupled filters (orthogonal to any importance criterion), and learned channel-selection [22] or prune-plus-distil [25] frameworks that fold selection into extra training—differ in kind from the one-shot importance scores we benchmark, and presuppose a differentiable pipeline that is absent in the gradient-free, post-quantization setting where SSA’s value lies. A head-to-head study of them on compact EEG backbones is a natural next step.

## 6. Conclusions

We evaluated ablation-based structural sensitivity analysis for pruning compact EEG CNNs against the standard gradient- and weight-based importance criteria, testing rather than assuming its advantage. Under a leakage-free, materialized, statistically controlled protocol spanning four datasets, two paradigms, two architectures, and a range of capacities, ablation (SSA) and first-order gradient (Taylor) importance are the two strongest criteria and, after fine-tuning, are formally equivalent on EEGNet (within ±1 accuracy point, pooled over four datasets) and—on ShallowConvNet—SSA is never worse than Taylor; both clearly outperform weight-magnitude and random pruning. The practical value of SSA is therefore not a more accurate ranking but its gradient-free formulation: we demonstrated that on a weight-quantized (8-bit) EEGNet—where the loss gradient, and thus Taylor importance, is undefined on the quantized graph used here—SSA still prunes effectively, extending structured pruning to the gradient-free-but-editable regime characteristic of embedded BCI deployment. Two design findings also generalize across our datasets: for final accuracy, the pruning schedule (per-layer over global) and the use of fine-tuning matter more than the choice among strong criteria—once fine-tuning is applied, the SSA/Taylor difference vanishes while per-layer scheduling keeps a consistent edge—and setting a network large then pruning with fine-tuning recovers the accuracy of a directly trained smaller model. Future work includes transfer of the per-layer width recipe across subjects (the ranking itself being subject-specific), additional architectures and an SSVEP paradigm, comparison against more recent structured-pruning methods, combining SSA-guided pruning with full quantization and knowledge distillation, and an on-device benchmark on embedded BCI hardware (memory, latency, energy).

## Figures and Tables

**Figure 1:**
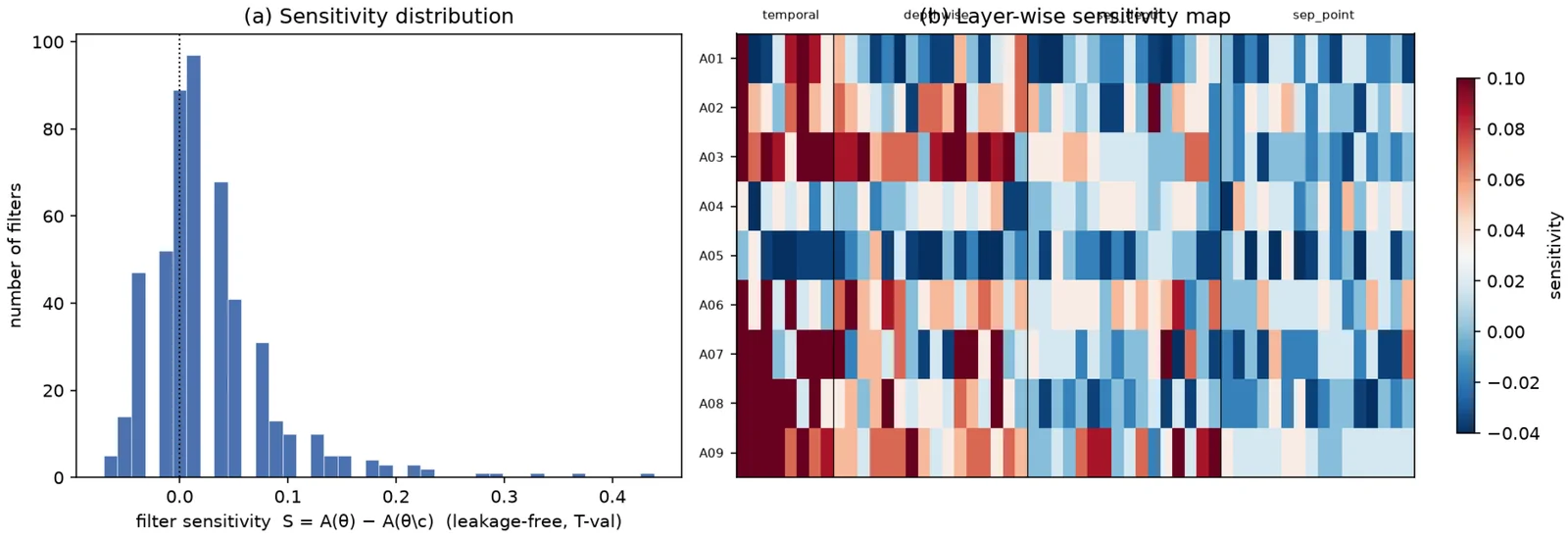
Leakage-free filter sensitivity (T-val): (**a**) distribution; (**b**) per-subject × filter map.

**Figure 2:**
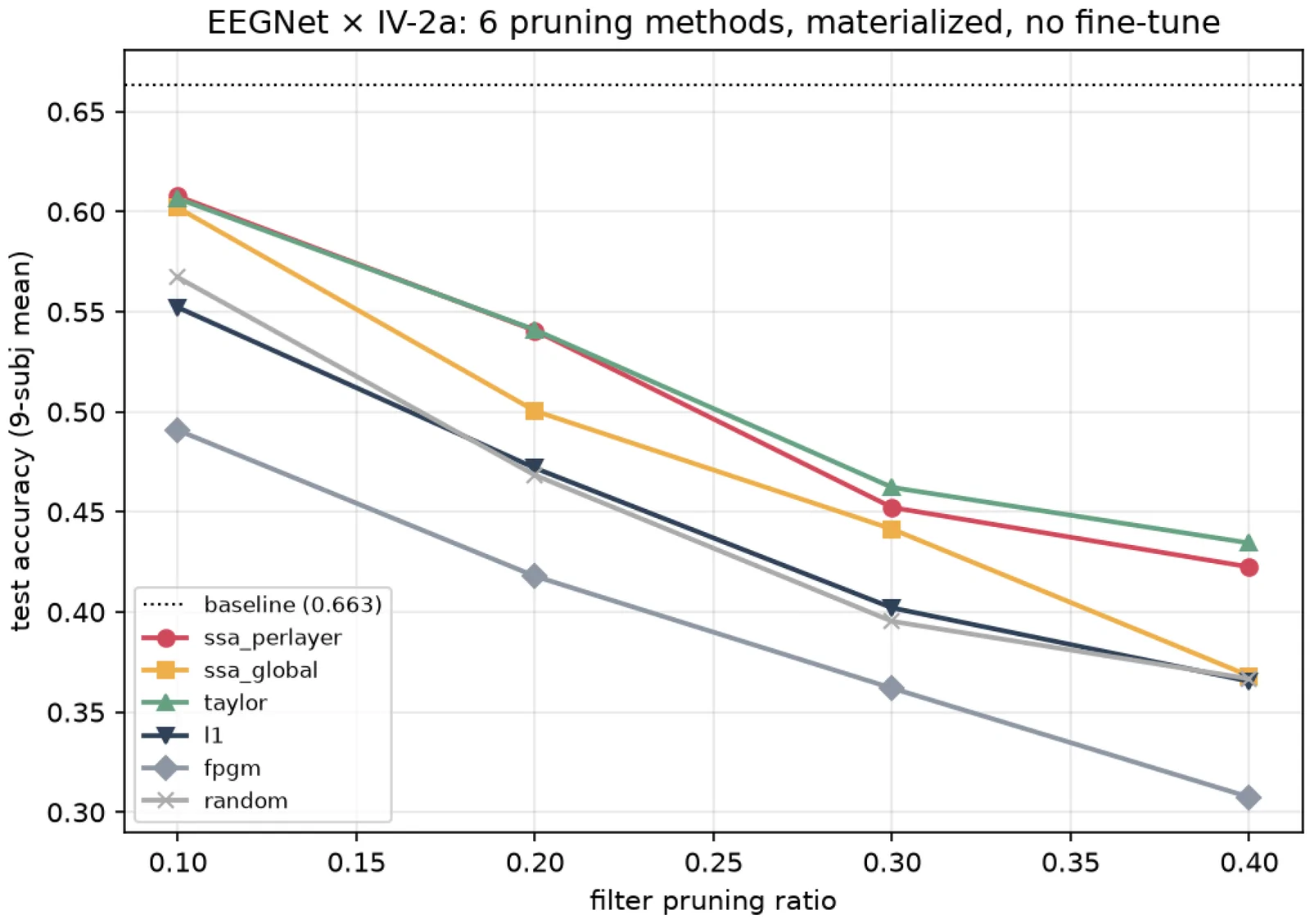
Six-method accuracy vs. pruning ratio (EEGNet × IV-2a, materialized, no-FT).

**Figure 3:**
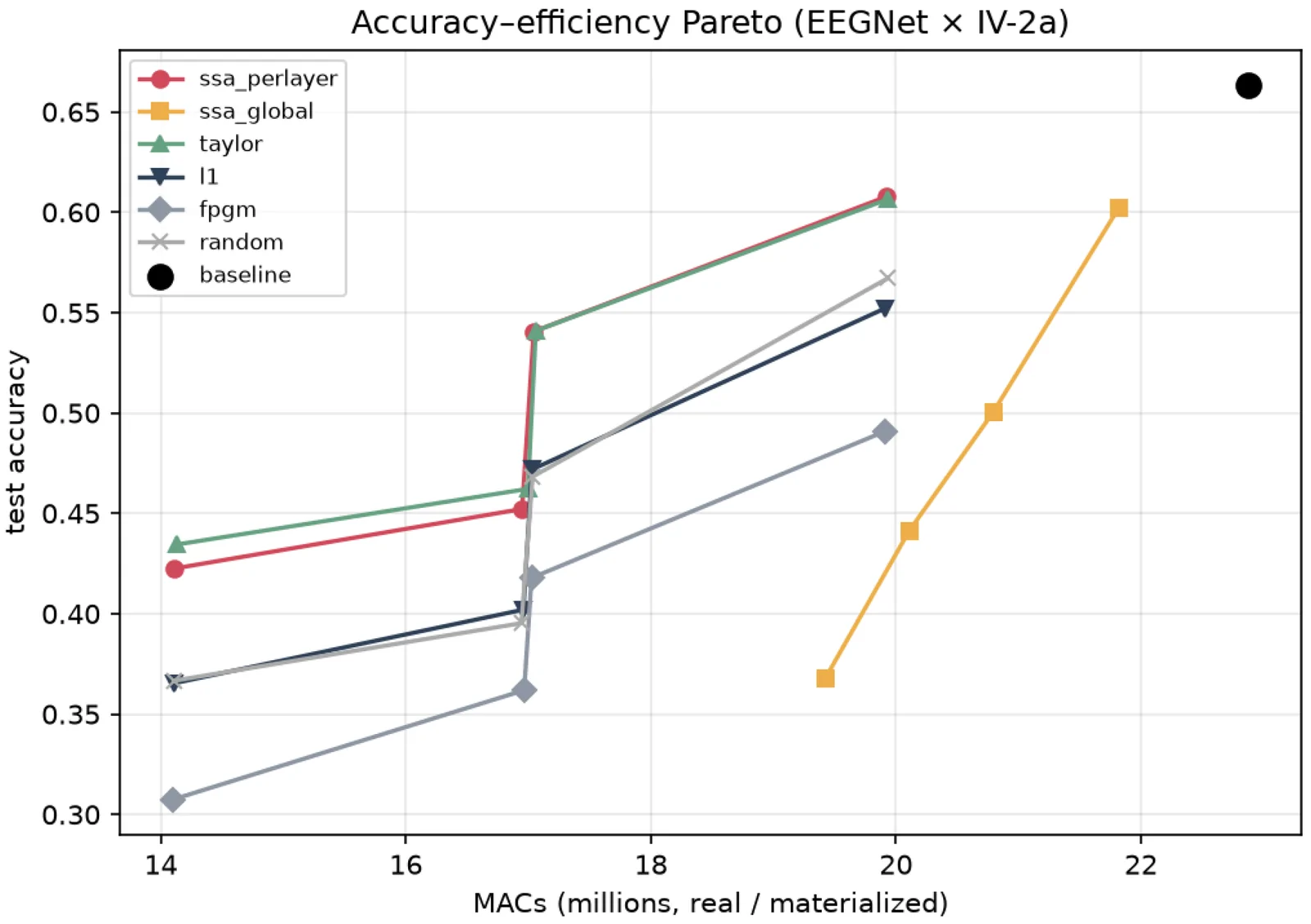
Accuracy vs. real (materialized) MACs.

**Figure 4:**
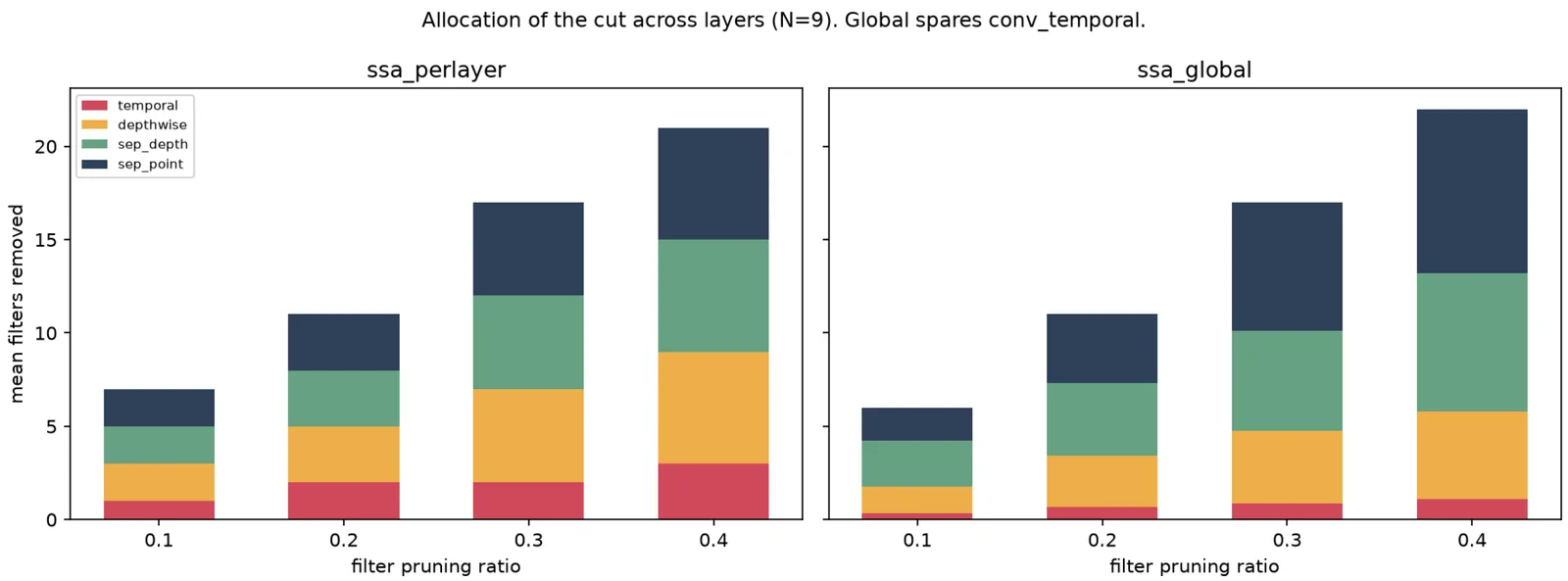
Per-layer vs. global allocation of the cut (N=9).

**Figure 5:**
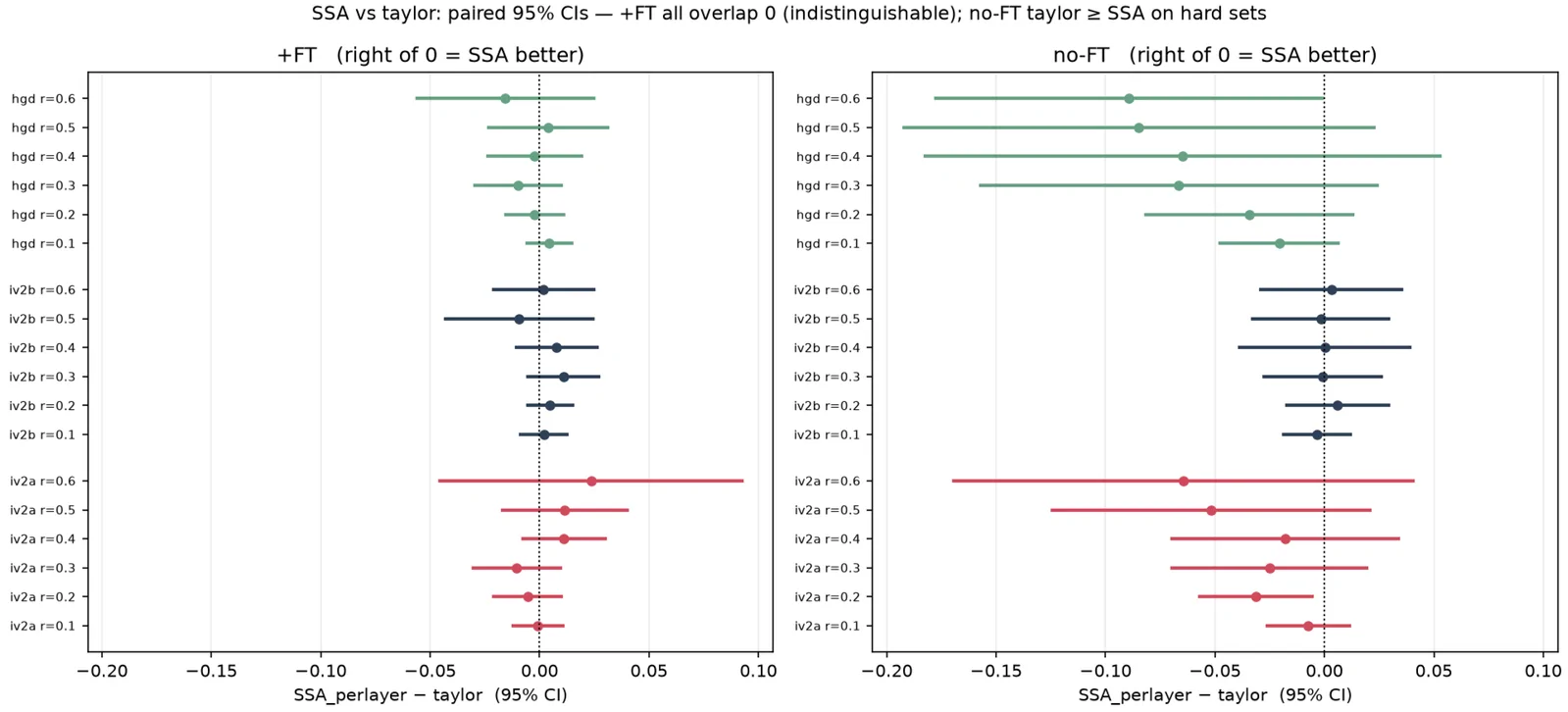
SSA–Taylor paired 95% CIs per dataset × ratio (+FT **left**, no-FT **right**). Each colour denotes a dataset; a point is the mean paired SSA–Taylor accuracy difference and the horizontal bar its 95% CI; the dashed vertical line marks zero difference (SSA equal to Taylor).

**Figure 6:**
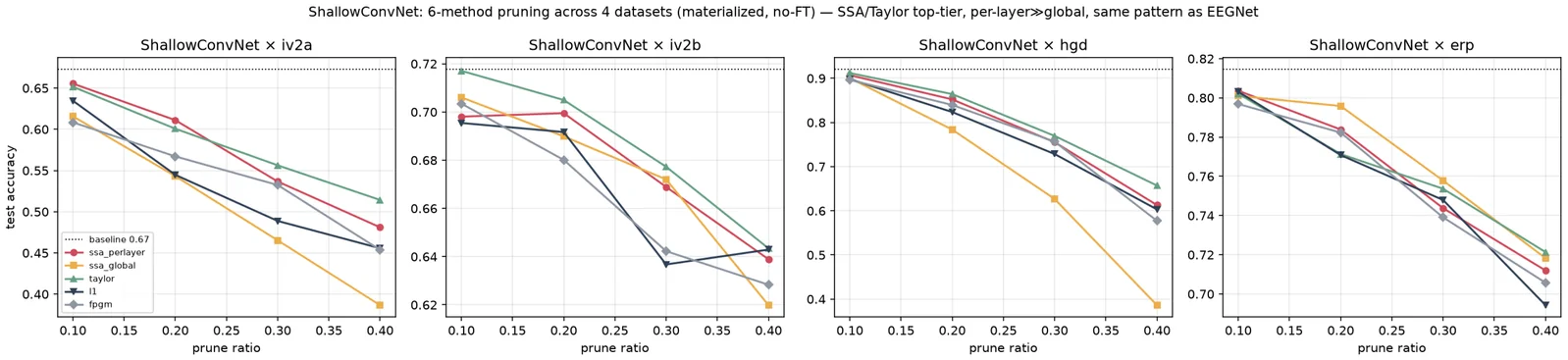
ShallowConvNet six-method pruning across four datasets.

**Figure 7:**
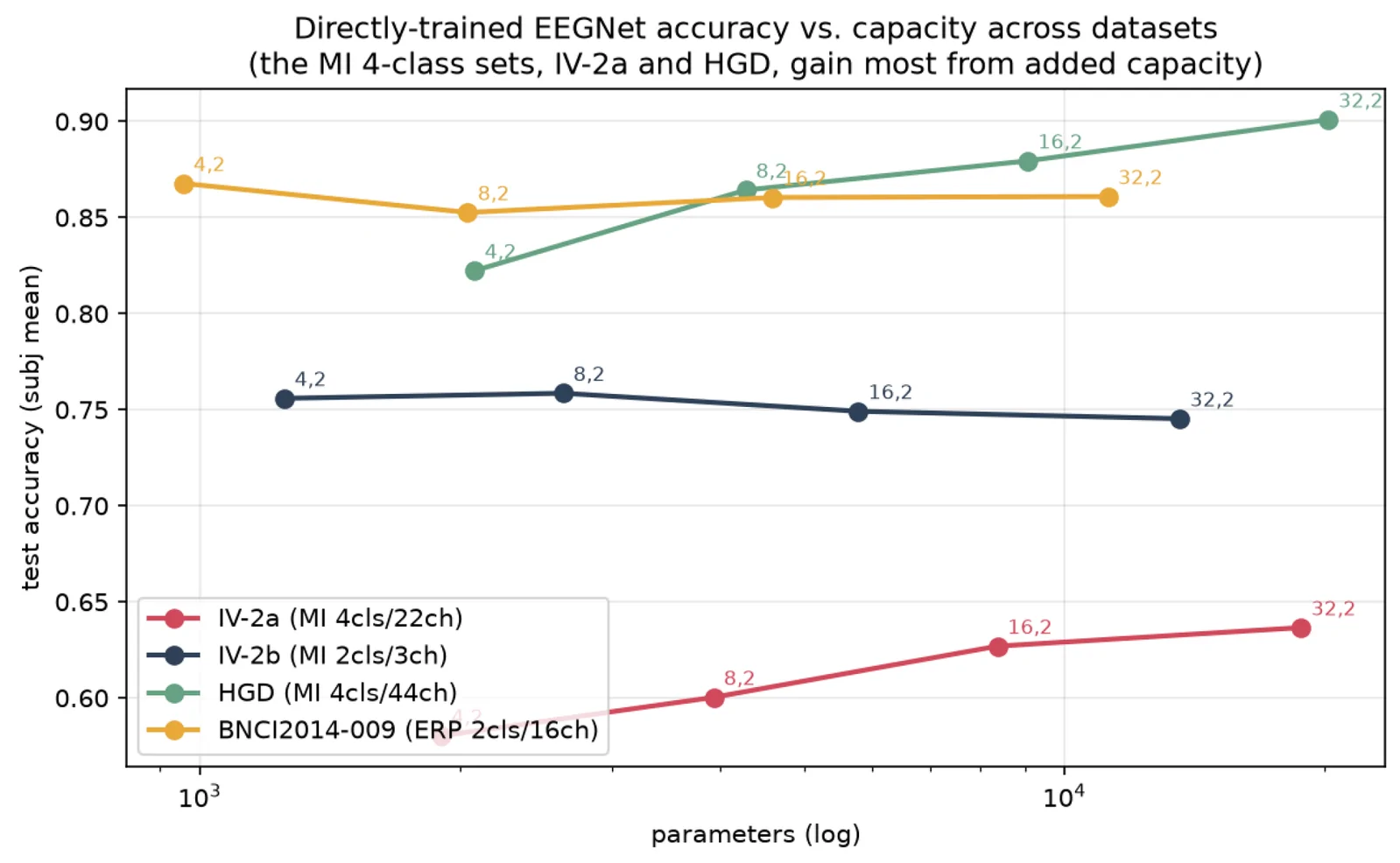
Directly trained accuracy vs. capacity across datasets.

**Figure 8:**
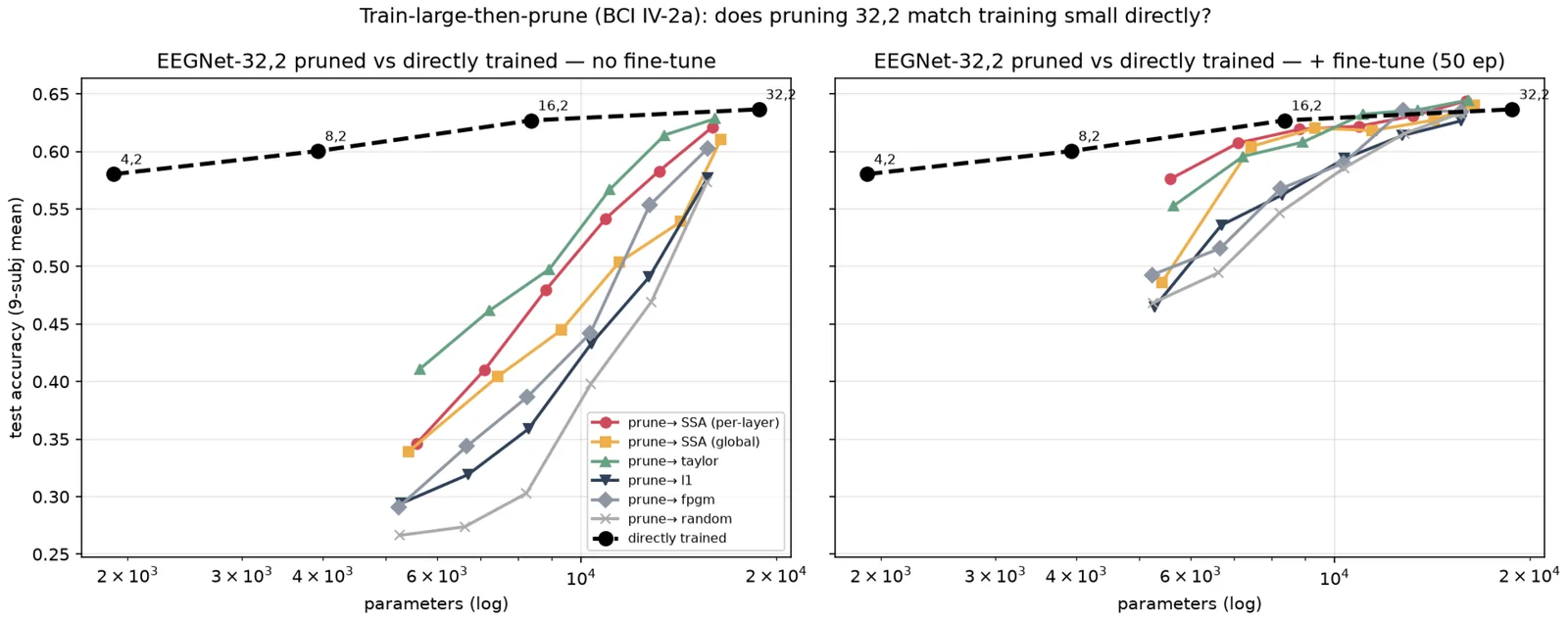
(IV-2a) EEGNet-32,2 pruned vs. directly trained smaller model.

**Figure 9:**
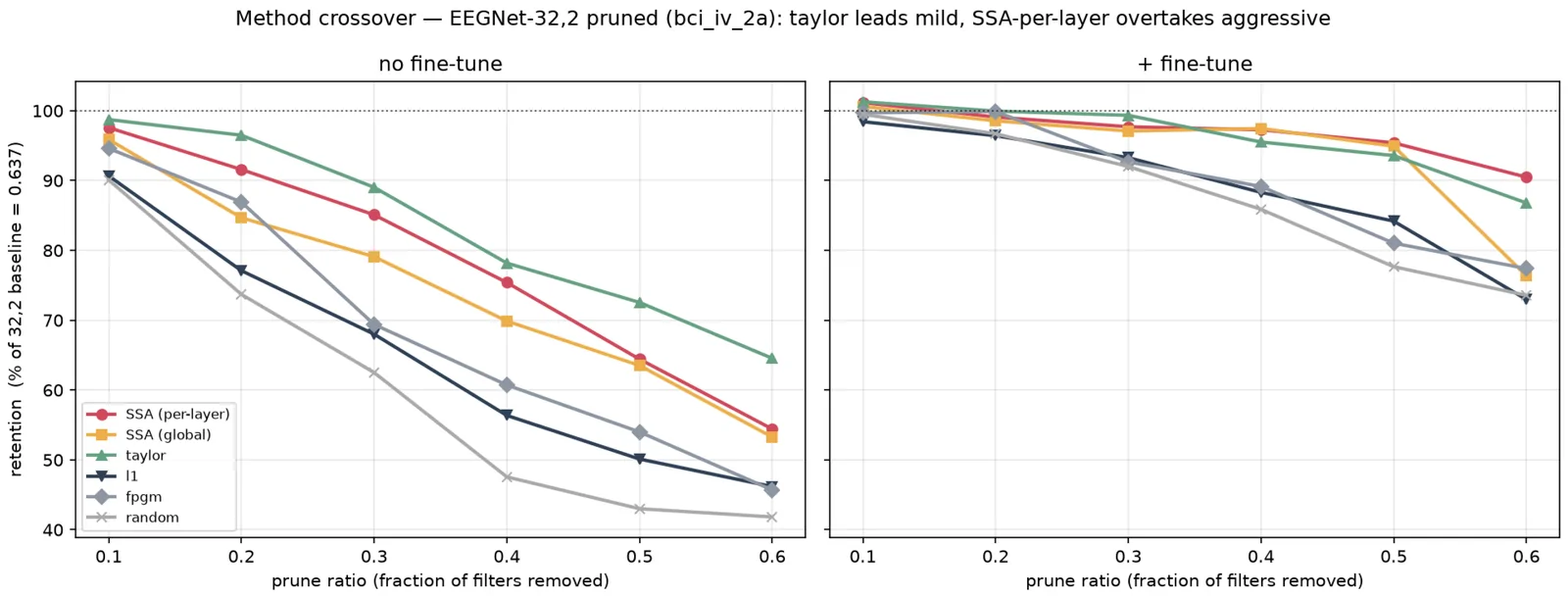
(IV-2a) Retention vs. ratio per method (±FT). The horizontal dashed line marks the unpruned baseline accuracy.

**Figure 10:**
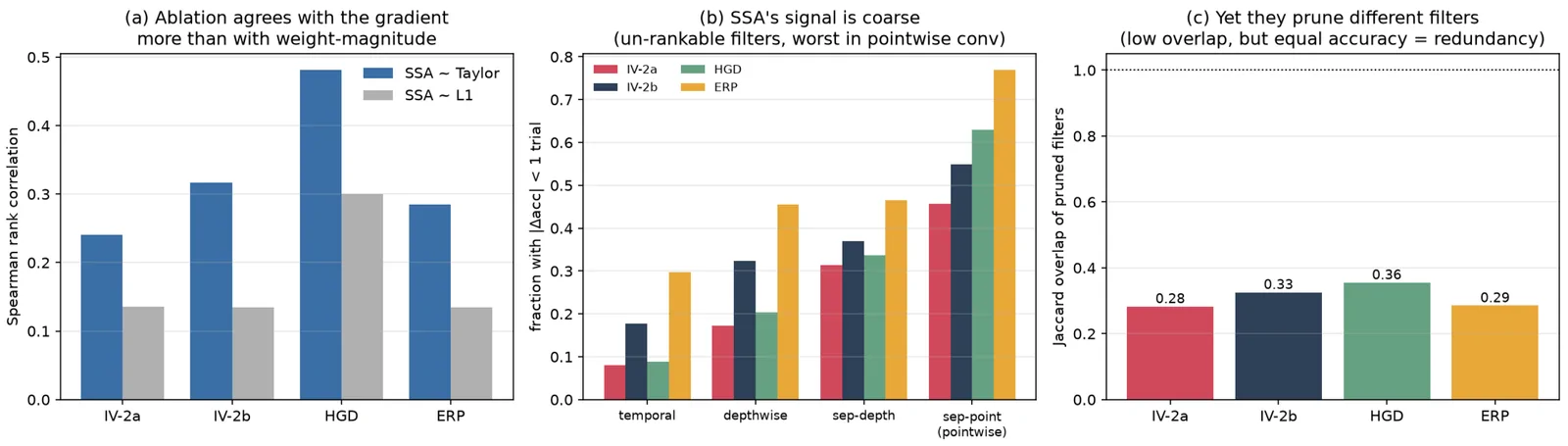
Anatomy of the SSA–Taylor parity (four datasets, EEGNet-32,2 source, scoring only). (**a**) SSA correlates about twice as strongly with the Taylor gradient as with weight-magnitude L1, so Taylor is the closer surrogate to direct ablation. (**b**) A large fraction of filters change validation accuracy by less than one trial and are thus unrankable by SSA, worst in the pointwise separable convolution. (**c**) SSA and Taylor prune only filter sets that overlap by ≈30%, yet reach identical fine-tuned accuracy: the accuracy is insensitive to *which* low-importance filters are removed; the dashed line in (**c**) marks complete overlap (Jaccard =1).

**Figure 11:**
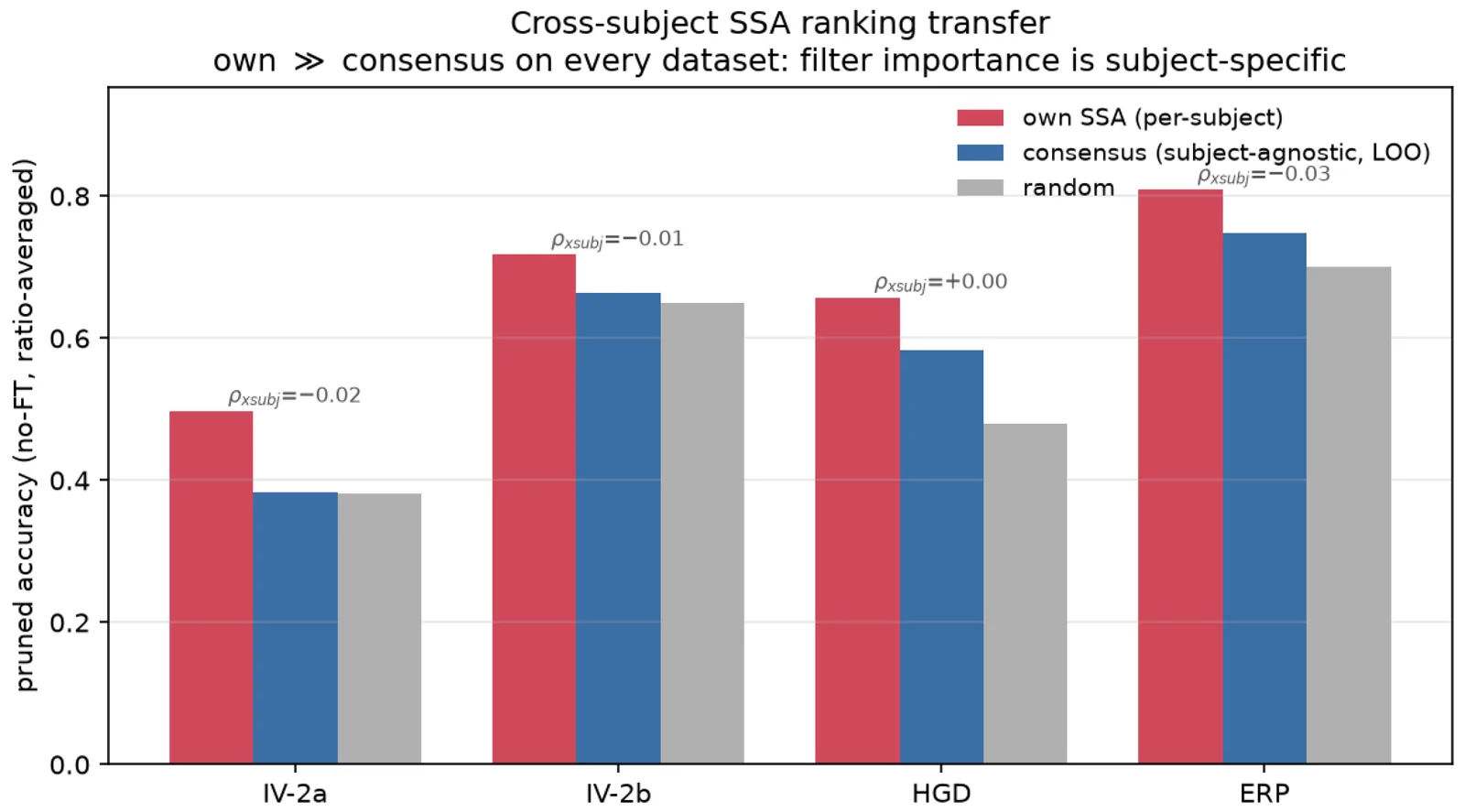
Cross-subject transfer of the SSA ranking (no fine-tuning, ratio-averaged). Each subject’s own ranking (red) prunes significantly better than a subject-agnostic leave-one-out consensus ranking (blue) on all four datasets; per-filter SSA scores are essentially uncorrelated across subjects ($\rho_{\mathrm{xsubj}}$, annotated). Filter importance is subject-specific, so the scoring cost cannot be amortized by reusing a ranking.

**Figure 12:**
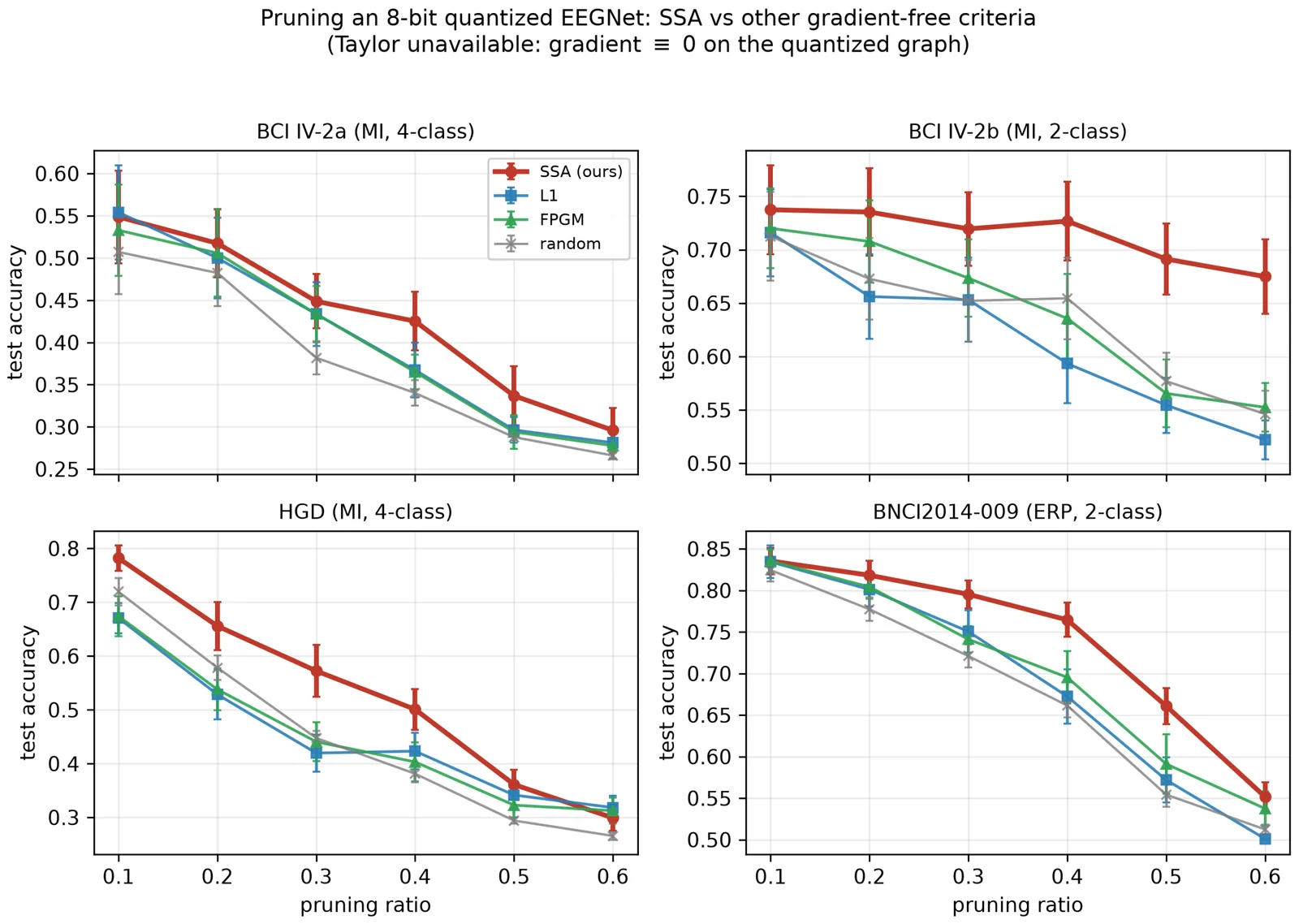
Pruning an 8-bit weight-quantized EEGNet with each gradient-free criterion, across four datasets and two paradigms (mean ± SEM). SSA attains the highest accuracy at essentially every pruning ratio; Taylor is absent because its gradient is identically zero on the quantized graph.

**Table 1:** Datasets.

Dataset	Paradigm	Classes	Channels	Subjects	Val Trials/Subj	EEGNet-8,2 Base
BCI IV-2a	MI	4	22	9	∼57	0.600
BCI IV-2b	MI	2	3	9	∼80	0.759
HGD	MI	4	44	14	∼176	0.864
BNCI2014-009	ERP/P300	2	16	10	∼77	0.853

**Table 2:** Per-dataset preprocessing and leakage-free evaluation protocol. Windows are relative to cue onset (motor imagery) or stimulus onset (ERP). No notch filter, baseline correction, or amplitude normalization is applied beyond the band pass. For the P300 set, non-target trials are subsampled to match the target count so that accuracy is a meaningful metric; the subsample is fixed across criteria.

Dataset	Rate (Hz)	Band Pass (Hz)	Window (s)	Normalization	Val/Test Split
BCI IV-2a	250	4–40	[0.5, 4.5] post-cue	None	T-session 80/20 (seed 42)/E-session
BCI IV-2b	250	4–40	[0.5, 4.5] post-cue	None	T-session 80/20 (seed 42)/E-session
HGD	250 (resampled)	4–40	[0.5, 4.5] post-cue	None	T-session 80/20 (seed 42)/E-session
BNCI2014-009	250	1–24	[0.0, 0.8] post-stimulus	None	T-session 80/20 (seed 42)/E-session

**Table 3:** Six-method benchmark accuracy (EEGNet × IV-2a, materialized, no-FT).

Method	0.1	0.2	0.3	0.4
FPGM	0.4907	0.4178	0.3619	0.3075
L1	0.5521	0.4718	0.4020	0.3654
Random	0.5671	0.4683	0.3953	0.3666
SSA-global	0.6019	0.5004	0.4414	0.3681
SSA-per-layer	0.6076	0.5405	0.4522	0.4225
Taylor	0.6065	0.5409	0.4622	0.4344

**Table 4:** SSA vs. Taylor paired statistics (EEGNet), ratio-pooled per dataset, ±FT. Difference is SSA–Taylor; lo/hi are 95% CI bounds. The per-ratio breakdown is shown in Figure 5.

Setting	Dataset	Mean	Lo	Hi	*t*-*p*	Wilcox *p*
+FT	IV-2a	0.0050	−0.0156	0.0255	0.593	0.652
+FT	IV-2b	0.0030	−0.0087	0.0147	0.567	0.652
+FT	HGD	−0.0036	−0.0213	0.0141	0.670	0.701
+FT	ERP	−0.0074	−0.0163	0.0015	0.138	0.131
no-FT	IV-2a	−0.0329	−0.0674	0.0016	0.059	0.039
no-FT	IV-2b	0.0006	−0.0185	0.0196	0.947	0.820
no-FT	HGD	−0.0600	−0.1234	0.0033	0.061	0.030
no-FT	ERP	−0.0207	−0.0360	−0.0055	0.026	0.027

**Table 5:** Criteria superiority: paired accuracy difference (winner − weaker criterion), pooled over four datasets (42 subjects), ratio-averaged. Both SSA and Taylor significantly beat every weight/redundancy-based and random criterion, with and without fine-tuning. *: p<0.05 (paired *t*-test).

Setting	Criterion	vs. L1	vs. FPGM	vs. Random
no-FT	SSA	+0.077 * [+0.049, +0.104]	+0.058 * [+0.035, +0.081]	+0.124 * [+0.101, +0.147]
	Taylor	+0.108 * [+0.078, +0.139]	+0.090 * [+0.065, +0.115]	+0.156 * [+0.128, +0.184]
+FT	SSA	+0.026 * [+0.017, +0.036]	+0.022 * [+0.012, +0.031]	+0.046 * [+0.036, +0.056]
	Taylor	+0.028 * [+0.018, +0.037]	+0.023 * [+0.014, +0.032]	+0.047 * [+0.036, +0.059]

**Table 6:** SSA vs. Taylor on ShallowConvNet (no-FT); difference is SSA–Taylor.

Architecture	Dataset	Mean Diff	CI Low	CI High	*t*-*p*	Result
ShallowConvNet	IV-2a	−0.0096	−0.0350	0.0157	0.406	ns
ShallowConvNet	IV-2b	−0.0093	−0.0356	0.0169	0.436	ns
ShallowConvNet	HGD	−0.0189	−0.0622	0.0244	0.364	ns
ShallowConvNet	ERP	−0.0013	−0.0387	0.0361	0.939	ns

**Table 7:** Ranking cost and applicability of importance criteria. Wall-clock time to score all filters once, on the train-large source model (EEGNet $F_{1}=32$, D=2; 224 prunable filters) for IV-2a, single GPU, mean of 3 subjects × 3 repeats. Absolute timings are hardware- and implementation-dependent; the intrinsic cost of SSA is O(n) forward evaluations (one per prunable filter), so it is slower than a single-backward-pass Taylor score by roughly two orders of magnitude, regardless of platform. “Needs gradient” = requires a usable loss gradient to form the ranking; “Quantized-applicable” = can rank filters on an 8-bit post-training-quantized model (Section 5.4).

Criterion	Ranking Time (ms)	Rel. to Taylor	Needs Gradient	Quantized-Applicable
SSA (ablation)	4949.7	139×	No	Yes
Taylor (first-order)	35.5	1.0×	Yes	No
L1-norm	2.09	0.06×	No	Yes
FPGM	9.70	0.27×	No	Yes
Random	0.06	—	No	Yes

**Table 8:** On the 8-bit quantized EEGNet, paired accuracy difference SSA —(each other gradient-free criterion), averaged over pruning ratios 0.1–0.6, per dataset and pooled. Positive favours SSA; * denotes p<0.05 (paired *t*-test). Taylor is excluded because its gradient is identically zero on the quantized graph.

Dataset	SSA − L1	SSA − FPGM	SSA − Random	*n*
BCI IV-2a (MI, 4-class)	+0.023 [−0.030, +0.077]	+0.027 [−0.006, +0.061]	+0.051 * [+0.006, +0.096]	9
BCI IV-2b (MI, 2-class)	+0.098 * [+0.046, +0.151]	+0.072 * [+0.033, +0.111]	+0.078 * [+0.046, +0.111]	9
HGD (MI, 4-class)	+0.078 [−0.001, +0.158]	+0.080 * [+0.032, +0.128]	+0.081 * [+0.029, +0.133]	14
BNCI2014-009 (ERP, 2-class)	+0.049 * [+0.008, +0.091]	+0.037 [−0.002, +0.076]	+0.063 * [+0.034, +0.092]	10
Pooled (all four)	+0.064 * [+0.031, +0.096]	+0.057 * [+0.035, +0.079]	+0.070 * [+0.048, +0.091]	42

## Data Availability

All datasets used in this study are publicly available. BCI Competition IV datasets 2a and 2b are available from the BNCI Horizon 2020 repository (http://bnci-horizon-2020.eu/database/data-sets, accessed on 12 July 2026); the High-Gamma Dataset and the BNCI2014-009 P300 dataset are accessible through the MOABB benchmarking framework (https://moabb.neurotechx.com/, accessed on 12 July 2026). The code and analysis scripts that support the findings of this study are available from the corresponding author upon reasonable request.
